# Growth Dynamic and Threshold Values for Spermicidal Effects of Multidrug-Resistant Bacteria in Extended Boar Semen

**DOI:** 10.3390/microorganisms11030788

**Published:** 2023-03-19

**Authors:** Anne-Marie Luther, Christina Beckermann, Thu Quynh Nguyen, Jutta Verspohl, Dagmar Waberski

**Affiliations:** 1 Unit for Reproductive Medicine, Clinic for Pigs and Small Ruminants, University of Veterinary Medicine Hannover, Foundation, Bünteweg 15, D-30559 Hannover, Germany; 2 Institute for Microbiology, University of Veterinary Medicine Hannover, Foundation, Bischofsholer Damm 15, D-30173 Hannover, Germany

**Keywords:** boar semen, antibiotic resistance, bacteria, semen preservation, artificial insemination

## Abstract

The aim of this study was first to examine the prevalence of bacteria-associated loss of sperm quality in samples from insemination centers during a seven-year semen monitoring program and, second, to investigate the growth dynamic of four different multidrug-resistant bacterial species and their impact on sperm quality during semen storage. A reduced sperm quality associated with bacterial contamination was found in 0.5% of 3219 of the samples from insemination centers. In samples spiked with *Serratia marcescens* and *Klebsiella oxytoca,* bacterial growth by six log levels was seen during storage at 17 °C, causing loss of sperm motility, membrane integrity, membrane fluidity, and mitochondrial membrane potential at >10^7^ CFU/mL (*p* < 0.05). Storage at 5 °C in the Androstar Premium extender efficiently inhibited their growth. *Achromobacter xylosoxidans* and *Burkholderia cepacia* showed limited growth up to two log levels at 17 °C and did not impair sperm quality. In conclusion, spermatozoa tolerate moderate loads of multidrug-resistant bacteria, and hypothermic, antibiotic-free semen storage effectively limits bacterial growth. The constant use of antibiotics in semen extenders should be reconsidered.

## 1. Introduction

Pig breeding is practiced worldwide with insemination of extended semen usually occurring after storage at 16 to 18 °C for several days [1]. Due to the natural occurrence of bacteria in the semen and possible bacterial contamination during the collection process, adding antibiotics to semen extenders is still regarded as mandatory to protect the sow’s health and sperm quality. As in hospitals, the continuous use of antibiotics and disinfectants in the semen collection area and in the semen processing laboratory favors the generation and spread of antimicrobial resistance in bacteria. This resulted in an increase in antibiotic use, including a shift towards “critically important” antibiotics for human use [2], such as macrolides (tylosin), polypeptides (polymixins), quinolones (enrofloxacin), cephalosporins (ceftiofur), or beta-lactam antibiotics [3]. Hence, the loss of efficiency and the ban of still-effective antibiotics in some countries, together with the adaptation of the One Health concept by the pig industry, have stimulated an intense search for alternative antimicrobial strategies [4,5]. The replacement of classical antibiotics in pig artificial insemination (AI) practice has not yet been achieved, although some promising attempts, for example, antimicrobial peptides [6,7], nanocomposites [8,9], or physicomechanical removal of bacteria, are being tested [10,11]. Typically, the majority of bacteria in boar semen are gram-negative opportunistic pathogens, which do not pose a threat to the sow’s health but may affect shelf-life during in vitro semen storage. It should be noted that the impact on sperm vitality depends on the bacterial species and the microbial load. Several studies have demonstrated that a bacterial load below 10^6^ CFU/mL with several mesophilic bacterial species, e.g., *Escherichia (E.) coli, Clostridium perfringens, Pseudomonas aeruginosa, Enterobacter cloacae*, does not affect sperm quality in vitro [12,13,14,15]. A litter size reduction was reported in the presence of more than 3.5 × 10^3^ CFU *E. coli* and a mixture of further aerobic gram-negative bacteria, which was strongly related (r = −0.87; *p* < 0.01) to the agglutination grade in the semen sample [16]. Routine quality control with an aerobic culture of extended porcine semen samples revealed bacterial loads > 10^2^ CFU/mL in 15 to 78% of screened samples [17,18,19,20,21], which mostly remained unnoticed in AI practice because neither sperm quality, nor fertility, nor sow health were affected. Field investigations in North America [17] and our own screenings of AI portions in Europe indicate that semen samples with a high degree of agglutination, low motility, and decreased longevity were most frequently contaminated with multidrug-resistant opportunistic pathogens. Among those, *Serratia (S.) marcescens*, *Klebsiella (K.) oxytoca*, and *Achromobacter (A.) xylosoxidans* were predominantly isolated in several field studies [21,22,23] and in our spermatology reference andrology laboratory. Their presence in low-quality samples enforces the overuse of antibiotics by aiming to completely eradicate virtually all bacteria as a preventive measure. To date, the dimension of the problem associated with bacteria-induced loss of sperm quality is unclear because published data on the incidence in routinely produced semen portions and systematic studies with field isolates of multi-resistant bacteria are lacking.

The aim of the present study was first to analyze the prevalence of bacteria in relation to sperm quality in routinely produced semen portions over a period of seven years. The second aim was to examine the growth dynamic of four different multidrug-resistant bacteria in extended boar semen, and their effect on sperm quality during long-term storage. A further objective was to test whether a reduction in the storage temperature from 17 to 5 °C could inhibit the growth of multidrug-resistant bacteria in semen extenders. Overall, the present study is aimed to stimulate concepts for a reduction in antibiotic use without impairing the biosecurity of extended boar semen.

## 2. Materials and Methods

### 2.1. Field Data Analysis

The aim was to examine the frequency of low-quality semen samples in association with bacterial contamination in commercial semen doses produced by 26 AI centers. For this, a retrospective analysis of field-monitoring data from random semen samples (n = 3219) over a seven-year period was performed. During a seven-year period (2016–2022), annually between March and June, an external semen quality control was conducted with the participation of 21 to 26 European AI centers (Germany, n = 16 to 21; Austria, n = 3; Switzerland, n = 2) as members of the Association for Bioeconomy Research (FBF e.V., Bonn, Germany). From each AI center, 18 to 20 randomly selected doses of freshly extended semen from different boars were shipped on the day of semen collection in insulated transport boxes to the spermatology reference laboratory at the University of Veterinary Medicine Hannover. Upon arrival the next day, the temperature was controlled and only semen samples with 17 ± 2 °C were examined. Samples were then stored at 17 °C in the dark. Semen analysis comprised sperm motility up until 72 h, presence of agglutination, morphology, and membrane integrity. Low-quality samples, defined as samples with motility < 70% at 72 h, agglutination grade > 2 (i.e., >20% agglutinated spermatozoa), or deteriorated sperm membranes in the 90th percentile of the preceding year, and 10 further random samples with high sperm quality were microscopically screened at 1000× in phase contrast for the presence of bacteria after 120 to 168 h of storage. In positively screened semen portions, subsamples were transferred the Institute for Microbiology at the University of Veterinary Medicine Hannover for aerobic culture on blood agar and subsequent identification of the bacteria with MALDI-TOF MS (microFlex LT, Bruker Daltonic, Bremen, Germany) with the software Biotyper (Bruker Daltonic, Server Version 4.1.100).

### 2.2. Experiments

#### 2.2.1. Chemicals and Media

Chemicals of analytical grade were purchased from Sigma-Aldrich Productions GmbH (Steinheim, Germany), Enzo Life Sciences GmbH (Lörrach, Germany), Thermo Fisher Scientific Inc. (Waltham, MA, USA), Carl Roth GmbH & Co. KG (Karlsruhe, Germany), Oxoid Deutschland GmbH (Wesel, Germany), Merck KGaA (Darmstadt, Germany), and Beckman Coulter GmbH (Krefeld, Germany). The Beltsville Thawing Solution (BTS) extender consists of 205 mM glucose, 20.4 mM Na_3_C_6_H_5_O_7_, 10.0 mM KCL, 15 mM NaHCO_3_, and 3.36 mM EDTA [24]. Androstar Premium was received from Minitüb GmbH (Tiefenbach, Germany). All extender media were sterile-filtered before use.

#### 2.2.2. Semen Collection and Processing

Eight sexually fertile boars (1 to 5 years of age) of different breeds (Landrace, Pietrain, and Duroc/Pietrain) were used as semen donors. Boars were housed on straw in individual pens at the Unit for Reproductive Medicine, University of Veterinary Medicine Hannover, and treated in accordance with the European Commission Directive for Pig Welfare. Semen collection was performed by trained technicians using the gloved-hand method at weekly intervals. All procedures were approved by the institutional Animal Welfare Committee of the University of Veterinary Medicine Hannover. Entire ejaculates without the bulbourethral gland secretion, which fulfilled the standards for semen use in artificial insemination, were used for the experiments. Semen was extended with pre-warmed (35 °C) BTS or Androstar Premium extender containing 0.25 mg/mL gentamicin sulfate to a final volume of 100 mL and a sperm concentration of 20 × 10^6^ sperm/mL. Samples were kept at room temperature for 90 min, after which they were stored for 72 h in the dark at +17 °C.

#### 2.2.3. Microbiology

##### Bacterial Inoculation of Samples

Four multidrug-resistant bacterial species, *A. xylosoxidans*, *B. cepacia*, *K. oxytoca*, and *S. marcescens*, were isolated from commercial semen portions and stored at −80 °C. Susceptibility tests are shown in Supplementary Table S1. At 24 h before inoculation, the bacterial isolates were cultured on Columbia agar with sheep blood (Oxoid Deutschland GmbH, Wesel, Germany) and incubated overnight at 37 °C. Colonies were diluted in a 2 mL extender and bacterial concentrations were adjusted after density photometry (SDM5, Minitüb GmbH, Tiefenbach, Germany). Extended semen samples or extenders were spiked with the bacterial solutions to a final concentration of approximately 5 × 10^3^ CFU/mL. Immediately after inoculation (0 h), the bacterial counts in extenders or extended semen samples were determined.

##### Bacterial Count

The total bacterial colony-forming units per milliliter (CFU/mL) was determined from a 10-fold serial dilution prepared in phosphate-buffered solution (PBS) ranging from 10^−1^ to 10^−10^. A volume of 100 μL of each dilution was plated on Columbia agar with sheep blood (Oxoid Deutschland GmbH) and incubated for 24 to 48 h at 37 °C under aerobic conditions. Bacterial colonies were counted, and total bacterial numbers were calculated and expressed as colony-forming units per milliliter (CFU/mL).

#### 2.2.4. Measurement of pH

The pH in the samples spiked with bacteria was measured with two types of pH test strips (Landgraf Laborsyteme GmbH, Langenhagen, Germany): pH 4.0–7.0, resolution 0.3 pH unit and pH 6.4–8.0, resolution 0.2 pH unit. The pH of the unspiked samples (controls) was measured with pH strips 6.4–8.0 and, additionally, with an electronic pH meter (SenTix 41/pMX 3000, WTW, Weilheim, Germany).

#### 2.2.5. Spermatology

##### Computer-Assisted Semen Analysis

The percentage of motile sperm was measured with the computer-assisted semen analysis (CASA) system AndroVision^®^ (Version 1.2, Minitüb GmbH), as described previously [25]. It was equipped with a TV adapter (60-C 1” 1.0×, Carl Zeiss Microscopy GmbH, Jena, Germany), a digital camera (acA2440–75uc, Basler AG, Ahrensburg, Germany), and an automated microscope warming stage. Aliquots were prewarmed under air at 38 °C for 30 min in a water bath and then measured in a 20 µL “Leja” chamber (Leja Products B.V., Nieuw Vennep, The Netherlands). At least 500 spermatozoa were recorded with a rate of 30 pictures per 0.5 s. A spermatozoon was considered “motile” when its curved-line velocity was higher than 24 µm/s and its amplitude of lateral head displacement was higher than 1 µm.

##### Assessment of Sperm Agglutinations

Five µL of extended semen samples was examined under a phase contrast microscope (Carl Zeiss Microscopy GmbH) with 200× magnification (ocular 10×, objective 20×, phase 2). The degree of agglutination was scored based on the estimated percentage of agglutinated sperm in at least three different fields: 0 = 0 to 5%, 1 = less than 20%, 2 = 20 to 40%, 3 = 40 to 60%, 4 = 60 to 80%, and 5 = 80 to 100%.

##### Flowcytometry

A flow cytometer (Cyto Flex, Beckman Coulter GmbH, Krefeld, Germany) equipped with three lasers (488 nm, 50 mW; 638 nm, 50 mW; 405 nm, 80 mW) was used. The gating was performed with CytExpert 2.4 Software (Beckman Coulter GmbH). Non-DNA-containing particles were identified by negative Hoechst 33342 stain and were excluded from analysis. At least 10,000 individual spermatozoa per sample were evaluated.

The integrity of sperm plasma membranes and acrosomes was assessed as previously described [25]. Briefly, semen subsamples were stained in final concentrations with 1.3 µmol/L Hoechst (H) 33342, 1.5 µmol/L propidium iodide (PI), and 2 µmol/L fluorescein conjugated peanut agglutinin (FITC-PNA). H 33342 was detected on fluorescence detector PB-450 (450/45 nm BP), FITC-PNA on FITC (525/40 nm BP), and PI on ECD (610/20 nm BP). Spermatozoa with intact membranes were negative for the stains of PI and FITC-PNA.

The sperm membrane fluidity was assessed with Merocyanine (M) 540 in viable (YO-PRO-1 negative) sperm as described by [26] with slight modifications. Briefly, semen subsamples were stained in final concentrations with 0.5 µmol/L H 33342, 0.004 µmol/L YO-PRO-1, and 0.108 µmol/L M 540. Signals were detected using filters 450/45 BP (H 33342), 525/40 BP (Yo-Pro 1), and 585/42 BP (M540). Viable spermatozoa with low membrane fluidity were negative for YO-PRO-1 and negative for M540.

The mitochondria membrane potential (MMP) was assessed with JC-1 (Enzo Life Sciences GmbH, Lörrach, Germany) in viable (PI-negative) spermatozoa, as previously described [25]. Briefly, semen subsamples were stained in final concentrations with 0.08 µmol/L H 33342, 0.08 µmol/L JC-1, and 0.22 µmol/L PI. Signals were detected using filters 450/45 BP (H 33258), 525/40 BP (JC-1 monomer), 585/42 BP (JC-1 aggregate), 450/45 BP (H 33342), and 610/20 nm BP (PI). Viable spermatozoa with a high mitochondrial membrane potential were negative for PI with JC-aggregates.

#### 2.2.6. Experimental Design

##### Experiment 1: Growth Dynamic of Resistant Bacteria and Sperm Quality

The aim was to monitor the growth dynamic of typical multidrug-resistant bacteria in boar semen portions and to identify threshold values for CFU in relation to the semen storage time for spermicidal effects. For this, semen extended in BTS with 0.25 mg/mL gentamicin was spiked with one of four different gentamicin-resistant bacterial species from commercial semen portions, i.e., *S. marcescens (*n = 6 samples, one per boar), *K. oxytoca* (n = 8 samples, one per boar), *A. xylosoxidans* (n = 8 samples, one per boar), and *B. cepacia* (n = 8 samples, one per boar), at three different initial bacterial counts (S1: ~10^2^ CFU/mL; S2: ~10^3^ CFU/mL; S3: ~10^4^ CFU/mL). Samples not spiked with bacteria served as control. Bacterial growth was evaluated during storage at 17 °C and sperm quality, i.e., sperm motility, agglutination, and membrane integrity, was compared with unspiked controls. The mitochondrial membrane potential and membrane fluidity were assessed in viable sperm in samples spiked at 10^3^ CFU/mL with *S marcescens* (n = 7 samples, one per boar), *K. oxytoca* (n = 10 samples from 8 boars), *A. xylosoxidans (*n = 10 samples from 8 boars), or *B. cepacia* (n = 10 samples from 8 boars) and compared with controls.

##### Experiment 2: Bacterial Growth in Semen Extenders at 17 °C and 5 °C

The aim was to test whether a previously identified antimicrobial acting extender for semen storage at 5 °C (Androstar Premium; [27]) is effective to inhibit the growth of *S. marcescens* and *K. oxytoca*, which are the bacteria with the highest growth dynamic identified in Experiment 1. For this, the antibiotic-free extenders Androstar Premium (Minitüb GmbH) and BTS (Control) were spiked with *S. marcescens* with an initial bacterial count of ~10^4^ CFU/mL. Samples were stored for 144 h at 17 °C and 5 °C. The experiment was conducted in four replicates.

### 2.3. Statistical Analysis

Data were analyzed using IBM SPSS Statistics Professional (SPSS Inc., IBM, Armonk, NY, USA). Data were checked for normal distribution with the Shapiro-Wilk Test. To address the repeated measurements, the Friedman Test (XLSX) or Kruskal Wallis Test (XLSX) was performed. Pairwise comparisons were performed with the Wilcoxon Test and corrected by Holm Bonferroni. Measurements were considered significant when *p* < 0.05. Unless otherwise stated, data are presented as mean ± the standard error of the mean (SEM).

## 3. Results

### 3.1. Field Data Analysis

The results are presented in Table 1. From 3219 extended semen samples collected from 26 AI stations during a seven-year period, 0.5% of the samples were positively screened for bacterial contamination ≥ 10^6^ CFU/mL. In these samples, five different bacterial species were identified: *A. xylosoxidans*, *B. species*, *E. coli*, *K. oxytoca*, and *S. marcescens*. An association between bacteria contamination and low sperm quality was found in 0.1% of the semen samples.

### 3.2. Experiment 1: Growth Dynamic of Resistant Bacteria and Sperm Quality

**Figure 1:**
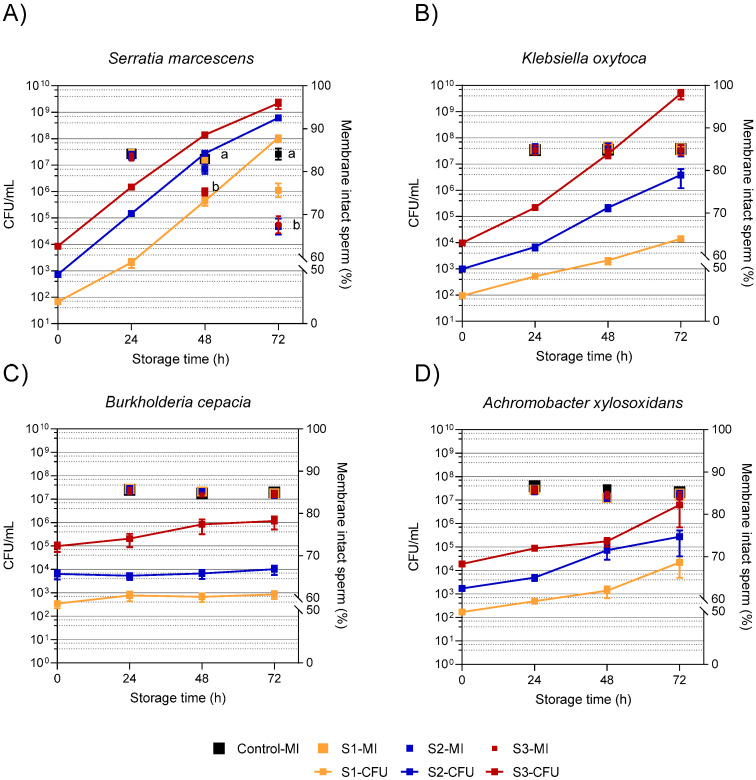
Bacterial growth (CFU/mL) and sperm membrane integrity in extended semen samples spiked with four different multidrug-resistant bacterial species at three different levels: S1, ~10^2^ CFU/mL; S2, ~10^3^ CFU/mL; and S3, ~10^4^ CFU/mL. The samples extended in Beltsville Thawing Solution were stored at 17 °C for 72 h. Sperm data are means and SEM (*S. marcescens*: n = 6; other bacteria: n = 8). Bacterial counts in controls were below the detection limit (<10^1^/mL) throughout storage. a, b: Different lowercase letters indicate differences between membrane-intact sperm (%, MI) at a given storage time point (*p* < 0.05). (**A**) *Serratia marcescens* (**B**) *Klebsiella oxytoca* (**C**) *Burkholderia cepacia* (**D**) *Achromobacter xylosoxidans*.

Figure 1 shows the bacterial growth after inoculation of semen samples at three different levels (S1–S3) and the effect on sperm membrane integrity during 72 h of semen storage. The growth of *S. marcescens* follows the increasing slope of typical exponential bacterial multiplication. At 24 h, when CFU were between 10^3^ and 10^6^/mL in all three sample types, sperm membrane integrity was at a similarly high level. In samples exceeding 10^7^ CFU/mL, the percentage of membrane-intact sperm declined (S2: 67 ± 5%, S3: 68 ± 5%; *p* < 0.05) regardless of the storage length (Figure 1A). *Klebsiella oxytoca* showed a rapid exponential growth only at the highest initial spiking level of 10^4^ CFU/mL (S3 reaching about 5 × 10^9^ CFU/mL after 72 h (Figure 1B). In samples with lower bacterial counts (S1, S2), the growth of *K. oxytoca* was slower and more linear. Sperm membrane integrity remained at a high level throughout storage and did not differ between the sample types. *Burkholderia cepacia* showed limited growth in extended semen, with a one-log-fold increase reaching 10^6^ CFU/mL in sample S3. Sperm membrane integrity was not affected during storage in all sample types (Figure 1C). *Achromobacter xylosoxidans* slowly increased, reaching between 10^3^ CFU/mL (S1) and 10^6^ CFU/mL (S3) after 72 h storage). Sperm membrane integrity did not differ between the sample types at any storage time (Figure 1D).

**Figure 2:**
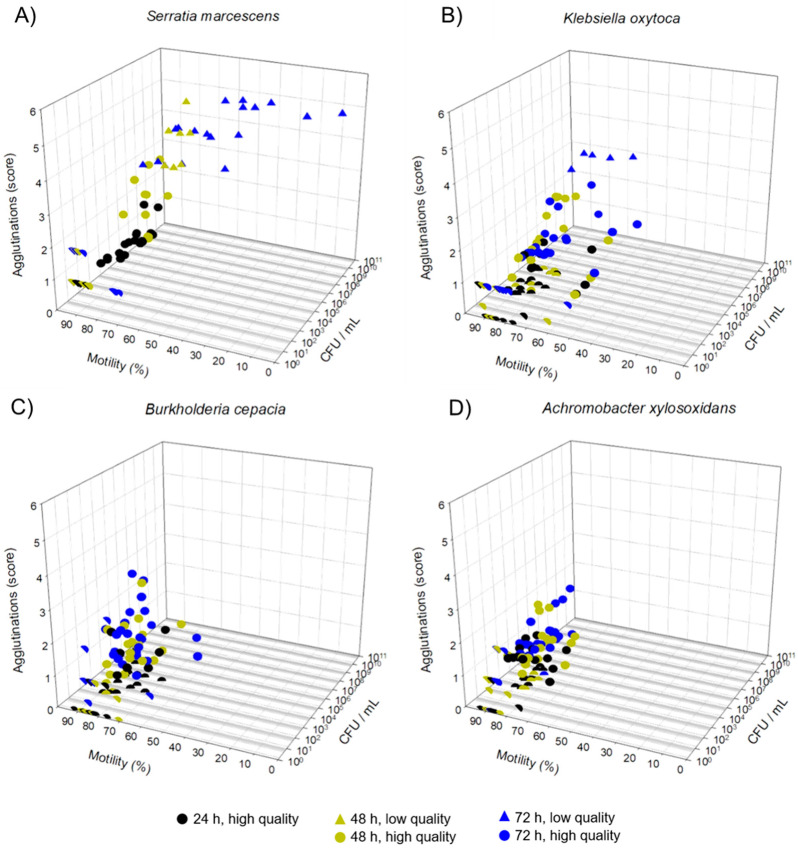
Three-dimensional plots showing the relation between bacterial counts (CFU/mL), sperm motility, and agglutination grade in extended semen samples spiked with four different multidrug-resistant bacterial species at ~10^3^ CFU/mL. The samples extended in Beltsville Thawing Solution were stored at 17 °C. Samples were analyzed at 24, 48, and 72 h, and the sperm quality was determined compared with unspiked controls. Samples (*S. marcescens*: n = 6; other bacteria: n = 7) were assigned as “low quality” if their difference to controls was > 10% for motility or > 2 for the agglutination score at a given storage time point. All other samples were grouped as “high quality”. (**A**) *Serratia marcescens* (**B**) *Klebsiella oxytoca* (**C**) *Burkholderia cepacia* (**D**) *Achromobacter xylosoxidans*.

Figure 2 shows the between bacterial load, sperm agglutination, and motility in a three-dimensional graph. The semen samples were assigned to high and low quality relative to their unspiked controls. In all samples with *S. marcescens* stored for 24 h, a high level of motility and a low level of agglutination (Score ≤ 2) were recorded. After 48 h, nine of 18 samples were assigned as low quality due to increased sperm agglutination. At 72 h of storage, 83% of samples were of low quality, this being caused by high agglutination and a loss of motility (Figure 2A). In samples inoculated with *K. oxytoca*, five samples (20.8%) were identified as low quality due to enhanced incidence of agglutination after 72 h when CFU were > 2 × 10^7^ mL (Figure 2B). Based on the degree of agglutination and motility, all samples spiked with *A. xylosoxidans* or *B. cepacia* maintained high quality. The proportion of low-quality samples in relation to the bacterial counts for four different bacterial species is shown in Table 2. Low-quality samples were detected starting with bacterial counts >10^7^ CFU/mL in some of the samples. When bacterial counts of *S. marcescens* were > 10^8^ CFU/mL, all samples (n = 21) were identified as low quality.

**Figure 3:**
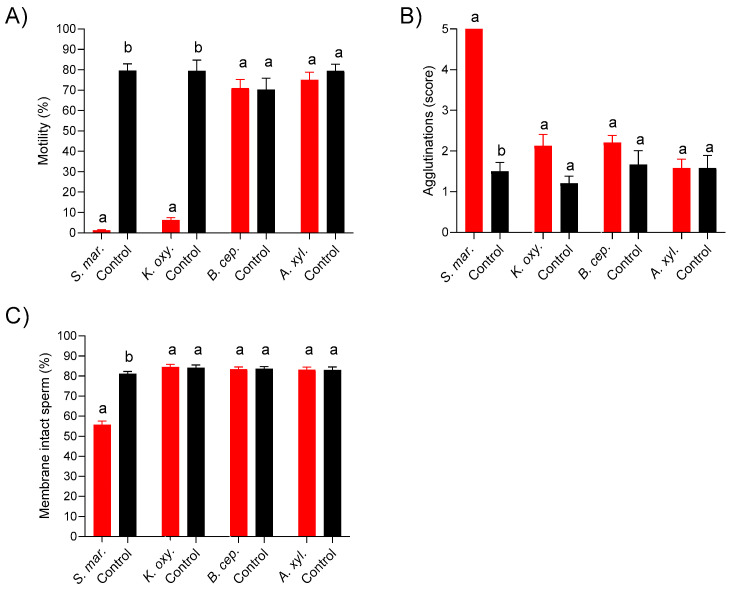
Sperm motility (**A**), agglutination (**B**), and sperm membrane integrity (**C**) in extended semen spiked with four different multidrug-resistant bacterial species at ~10^3^ CFU/mL and stored for 144 h. The samples extended in Beltsville Thawing Solution were stored at 17 °C. Data are means and SEM (*S. marcescens*: n = 6; other bacteria: n = 8). a, b: Different lowercase letters indicate differences between spiked samples and controls (*p* < 0.05).

Figure 3 shows the long-term effect (144 h storage time) of bacteria on the sperm quality traits of the four bacterial species in samples with the initial inoculation level ~10^3^ CFU/mL. In samples with *S. marcescens*, more than 90% of the sperm were agglutinated, which was accompanied by a dramatic loss of motility and membrane integrity (*p* < 0.05). Samples spiked with *K. oxytoca* revealed a strong loss of motility (*p* < 0.05), whereas agglutination and membrane integrity did not differ from the controls. The presence of *A. xylosoxidans* or *B. cepacia* did not affect the three sperm quality traits.

**Figure 4:**
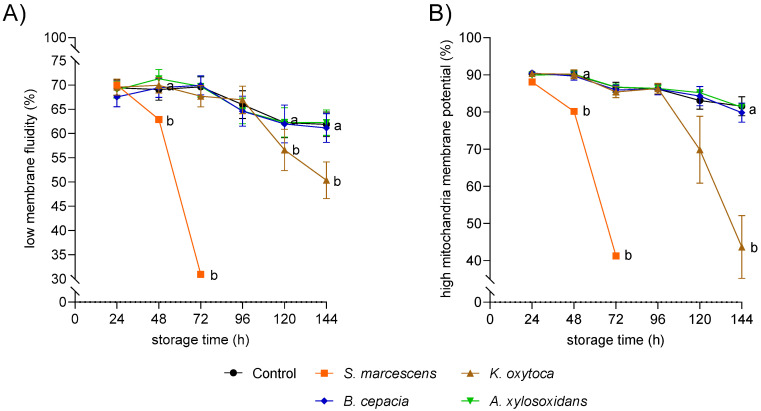
Viable sperm with low membrane fluidity (**A**) and viable sperm with high mitochondrial membrane potential (**B**) in extended semen spiked with four different multidrug-resistant bacterial species at ~10^3^ CFU/mL. The samples extended in Beltsville Thawing Solution were stored at 17 °C for 144 h. Data are means and SEM (*S. marcescens*: n = 7; other bacteria: n = 10). a, b: Different lowercase letters indicate differences between spiked samples and controls at a given storage time point (*p* < 0.05).

Figure 4 shows the membrane fluidity and mitochondrial membrane potential in viable spermatozoa of semen samples spiked with ~10^3^ CFU/mL of the four bacterial species. The percentage of viable spermatozoa with low membrane fluidity in samples spiked with *S. marcescens* decreased from 70.0 ± 0.01 at 24 h to 31.0 ± 0.03% at 72 h and differed from the controls (*p* < 0.05). Samples spiked with *K. oxytoca* showed lower values (*p* < 0.05) for membrane fluidity compared with the controls after 120 h of semen storage. During storage for 144 h, the sperm membrane fluidity of samples containing *A. xylosoxidans* or *B. cepacia* was not affected compared with the controls (Figure 4 A). The percentage of viable spermatozoa with high mitochondria membrane potential (hMMP) in samples with *S. marcescens* declined within 48 h of storage to 80.2 ± 0.02 and differed from the controls (90.0 ± 1.1, *p* < 0.05). There was a rapid strong decrease in sperm with hMMP to 41.2 ± 0.1 at 72 h (*p* < 0.05). In samples stored in the presence of *K. oxytoca*, the percentage of viable sperm with hMMP varied between samples at 120 h and decreased significantly at 144 h to 43.7 ± 8.4 (*p* < 0.05; Figure 4 B).

**Figure 5:**
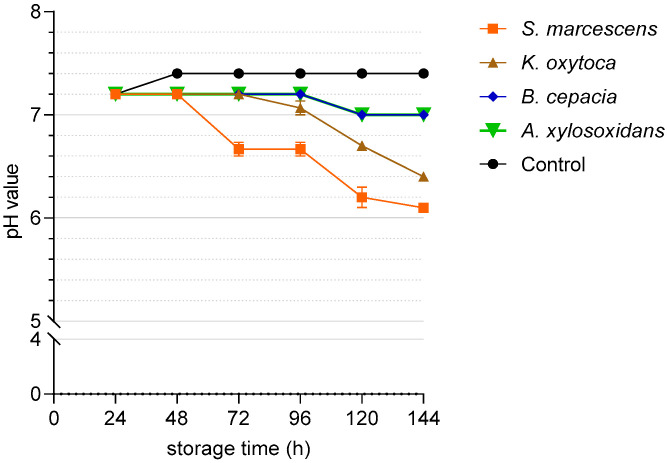
pH values in extended semen spiked with four different multidrug-resistant bacterial species at ~10^3^ CFU/mL. The samples extended in Beltsville Thawing Solution were stored at 17 °C for 144 h. Data are means and SEM (n = 3).

Figure 5 shows the pH in the semen samples spiked with ~10^3^ CFU/mL of the four bacterial species during 144 h storage. The pH decreased in samples with *S. marcescens* by 0.5 units to 6.7. at 72 h and dropped to 6.1 at 144 h. Samples containing *K. oxytoca* showed a slower acidification, reaching a pH of 6.4. at 144 h. In the presence of *A. xylosoxidans* or *B. cepacia*, the pH decreased by 0.2 units to 7.0 during 144 h of storage.

Taken together, the results of Experiment 1 demonstrate that the growth dynamics and the lower limit of bacterial concentration affecting sperm quality highly differ between the four tested bacterial species. For *S. marcescens*, spermatologically conspicuous samples started at bacterial counts of 10^7^ CFU/mL. For *K. oxytoca*, the spermicidal threshold shifted toward bacterial counts higher than 10^8^ CFU/mL. Due to the significantly reduced growth dynamics of *A. xylosoxidans* and *B. cepacia*, sperm-damaging bacterial counts were not achieved up to 144 h of storage.

### 3.3. Experiment 2: Bacterial Growth in Semen Extenders at 17 °C and 5 °C

**Figure 6:**
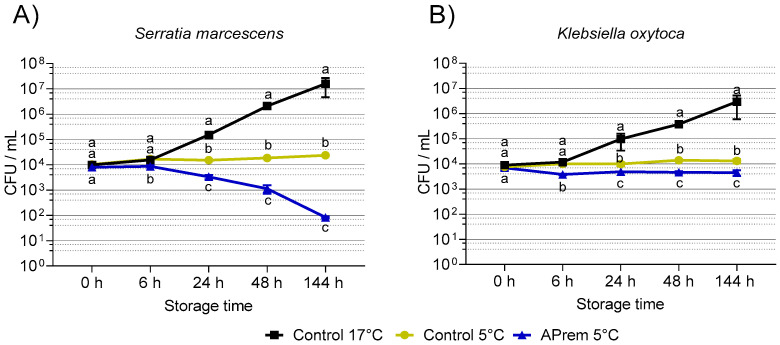
Growth of multidrug-resistant *S. marcescens* (**A**) and *K. oxytoca* (**B**) during storage for 144 h at 5 °C in BTS and Androstar Premium extender. Samples stored in BTS at 17 °C were used as controls. a–c: Different lowercase letters indicate differences between semen extenders at a given storage time point (*p* < 0.05).

Within the first 72 h at 17 °C, *S. marcescens* grew to 7.3 × 10^5^ ± 4.4 × 10^5^ CFU/mL in BTS, and to 1.4 × 10^5^ ± 6.2×10^4^ in Androstar Premium, starting with the initial spiking level of ~10^4^ CFU/mL. Figure 6 shows the growth of *S. marcescens* and *K. oxytoca* in two different extenders during storage at 5 °C compared with the positive control at 17 °C. Storage for 144 h at 5 °C showed lower bacterial counts (*p* < 0.05) for *S. marcescens* (Figure 6A) and *K. oxytoca* (Figure 6B) compared with storage at 17 °C. Growth inhibition of *S. marcescens* was more effective in Androstar Premium compared with BTS (*p* < 0.05), corresponding to an additive antimicrobial effect of storage temperature and extender type. At 144 h, the bacterial load of *S. marcescens* decreased from initially 10^4^ to less than 10^2^ CFU/mL. The extender-specific antimicrobial effect of Androstar Premium was less expressed for *K. oxytoca* compared with *S. marcescens.* The data indicate that storage at 5° C is effective to inhibit the growth of two multidrug-resistant bacteria displaying a fast growth at 17 °C in extended boar semen.

## 4. Discussion

Drug-resistant microbes are frequently detected in the bacterial screening of semen doses. However, the dose–response to sperm quality or fertility is often neglected. The present study demonstrates that the presence of <10^7^ CFU/mL of four multidrug-resistant bacterial species, i.e., *S. marcescens*, *K. oxytoca*, *B. cepacia*, and *A. xylosoxidans*, does not affect the quality of extended boar semen. The duration of sperm exposure to bacteria during semen storage seems to be of minor importance as long as the bacterial load remains under the critical value that affects sperm quality. The threshold value for bacterial load reported here is in line with observations of other opportunistic but less resistant bacteria, which have been frequently identified in boar semen [12,13,14,15]. In AI routine, the majority of semen is used within three days after semen collection, although long-term extenders enable semen storage for up to seven days [1]. Even with the high spiking dose (10^4^ CFU/mL), within 72 h of semen storage spermicidal, threshold values of 10^7^ CFU/mL were only reached with *S. marcescens* and *K. oxytoca*. The most rapid growth was seen for *S. marcescens*, which coincides with the short generation interval of 4.3 h observed in semen-free extender media at 16 °C [22] and field observations in commercial semen portions [12]. Similar to reports on other typical contaminants in boar semen [12,16,18], bacteria-induced loss of sperm quality was first expressed by an increase in sperm agglutinates and loss of motility, and thus it allows easy and fast detection in the routine analysis of stored samples. Bacteria-induced changes in sperm membrane fluidity and mitochondria membrane potential were only evident for *S. marcescens* and *K. oxytoca* after prolonged storage. The acidification of semen samples inoculated with *S. marcescens* or *K. oxytoca* may have contributed to the observed loss in sperm motility after longer storage when > 10^7^ CFU/mL was reached. It has been shown that the pH of the medium directly affects motility by a change in the inner-sperm pH through its effect on the flagellar beat frequency [28], resulting in a loss of motility when the pH is lowered from 7.2 to 6.2 [29].

Depending on the degree of bacterial counts and storage time of semen before use for insemination, particularly the occurrence of *S. marcescens* can have dramatic effects on sow fertility and the productivity of an AI center. Interestingly, in the present retrospective seven-year study, only 0.5% of 3219 randomly screened commercial semen doses were identified with bacteria-associated loss of sperm quality; among these, only one case was of *S. marcescens* and two cases were of *K. oxytoca*. It is important to note that all of the 26 AI centers participate continuously in a science-based quality control (QC) program conducted by the two national spermatology reference laboratories of the German Livestock Association (BRS; [30,31]). The application and control of hygiene concepts together with the prudent use of antibiotics in semen extenders [1] proved to be highly effective in reducing bacterial contamination and avoiding the generation of antimicrobial resistance [3,19].

The present study also shows that two other multidrug-resistant bacterial species, *A. xylosoxidans* and *B. cepacia*, were minor threats for sperm quality because of their slow growth in extended semen. Similar to *S. marcescens* and *K. oxytoca*, they seem not to impair sperm quality when CFU are below 10^7^/mL. Whether a higher load of these bacteria is sperm-damaging remains to be shown, as in the present study, these values were not reached, even with the initially highest load level of 10^4^ CFU/mL. It should be noted that such a high amount of a single bacterial species in freshly extended boar semen is rare. Clearly, boar semen may contain many other bacterial species than studied here, either from animal, environmental, or human sources. Among those, *Enterococcus species*, *Pseudomonas*, *Staphylococcus*, *Proteus*, and *E. col*i, occur most frequently [16,17,20,32,33]. In the present field data analysis, however, none of these bacteria were identified in low-quality semen samples, indicating that they either were absent or sensitive to the antibiotic in the semen extender or grew only slowly, so that spermicidal effects were not reached.

A further aim of the present study was to open new perspectives for limiting the growth of resistant bacteria, especially those of major concern for AI practice. We recently demonstrated that specific semen extenders may have an intrinsic antimicrobial activity by showing that the commercial long-term extender Androstar Premium acts in the absence of antibiotics against the typical bacterial flora in boar semen [27]. The present study, however, revealed that the antibacterial effect of this extender was insufficient to inhibit the growth of *S. marcescens* at the conventional storage temperature of 17 °C. In contrast, semen storage at 5 °C acted bacteriostatically for S. *marcescens* and *K. oxytoca*. The inhibition of bacterial growth was more effective in the Androstar Premium compared with the BTS extender, pointing to a synergistic effect of hypothermic storage and the extender type. In any case, semen storage in the traditional short-term extender BTS is not applicable because this extender does not protect against chilling injury [34]. Boar spermatozoa are highly susceptible to chilling injury, resulting in loss of motility, membrane damage, and disturbed capacitation [35,36,37]. Recent in vitro and in vivo field studies, however, demonstrated that the Androstar premium extender acts in a protective manner to cold-shock and thus allows hypothermic semen storage with high fertility and efficient control of commensal seminal bacteria even in the absence of antibiotics [26,38,39,40]. The present study demonstrates that the hypothermic semen storage in Androstar Premium is a practical tool for also controlling the growth of multidrug-resistant bacteria to counts that are not detrimental to sperm functionality.

The presence of residual bacteria in extended semen could even present an advantage over complete eradication by antibiotics. There is an initial understanding that the male reproductive microbiome might also act beneficially for fertility, for example, by influencing sperm competition or by immunogenic interaction with the female reproductive tract [41]. In boar semen, recently, 254 bacterial species were identified by high-throughput metagenomics [23], predominantly belonging to the phylum Proteobacteria, like the four bacterial species studied here. Their role in reproductive physiology is still largely unknown. From this perspective, it could be even unwise to completely eradicate all seminal microbes.

## 5. Conclusions

Multidrug-resistant bacteria, similar to many other isolated microbes in boar semen, are not detrimental to spermatozoa if their growth in extended semen is kept below 10^7^ CFU/mL. Acceptance of the prevalence of low to moderate bacteria loads and consideration of their potential benefit will be a large step forward to avoid the overuse of antibiotics and the associated generation of antimicrobial resistance in pig insemination. In the clinical setting, this would promote a sustainable porcine breeding management by lowering the risk of emerging multi-drug resistances in sow herds and entry of antibiotics into the environment. Given the high efficiency of hygiene concepts and QC programs, the preventive use of antibiotics with the highest relevance for human health in semen extenders seems inappropriate. Regular screening of AI portions for resistant microbes and the identification of the source of contamination is recommended. Concomitantly, emerging antimicrobial strategies that limit the multiplication of *S. marcescens* and *K. oxytoca* and, potentially, other fast-growing bacterial species to values below spermicidal thresholds, should be considered. In this sense, semen storage at 5 °C in an appropriate extender medium is an established practicable tool, especially if effective antibiotics are no longer available.

## Figures and Tables

**Table 1:** Sperm quality and bacterial isolations in a seven-year quality monitoring program using 3219 random semen portions from 26 European AI centers (18 to 20 samples from each AI center). Low quality was defined as total motility < 70%; agglutination score >2 or >5%; defective sperm membranes > 90th percentil of the preceding year.

Year	AI Centers (n)	Semen Samples (n)	High Quality, No Bacterial Isolation (n)	High Quality, Bacterial Isolation (n)	Low Quality, No Bacterial Isolation (n)	Low Quality, Bacterial Isolation (n)	Bacteria Type
2016	26	513	437	7 *	67	2	*B. species*, *E. coli*, *K. oxytoca*
2017	24	480	466	0	14	0	-
2018	24	479	424	0	55	0	-
2019	23	450	436	2 **	12	0	*A. xylosoxidans*, *S. marcescens*
2020	22	440	427	5 **	7	1	*K. oxytoca*
2021	22	437	396	0	41	0	-
2022	21	420	362	0	58	0	-
Sum		3219	2948 (91.6%)	14 (0.4%)	254 (7.9%)	3 (0.1%)	

* Two AI centers; ** One AI center.

**Table 2:** Incidence of low-quality samples in relation to bacterial species and bacterial counts (CFU/mL) in extended semen during storage for 72 h in Beltsville Thawing Solution. Semen samples were spiked with four different multidrug-resistant bacterial species at ~10^3^ CFU/mL and analyzed at 24, 48 and 72 h. Samples (*S. marcescens*: n = 6; other bacteria: n = 7) were assigned to “low quality” if the difference to controls was > 10% for motility or > 2 for the agglutination score at a given bacterial count level.

	Bacteria (All Samples/Low Quality Samples)			
Bacterial Count CFU/mL	*Serratia marcescens*	*Klebsiella* *oxytoca*	*Burkholderia cepacia*	*Achromobacter xylosoxidans*
	All (n)/Low (n)	All (n)/Low (n)	All (n)/Low (n)	All (n)/Low (n)
<10^6^	18/0	51/0	69/0	69/0
10^6^–10^7^	6/0	8/0	3/0	2/0
10^7^–10^8^	9/5	7/1	-	1/0
>10^8^	21/21	6/4	-	-

## Data Availability

The data presented in this study are available on request from the corresponding author.

## Supplementary Materials

The following supporting information can be downloaded at https://www.mdpi.com/article/10.3390/microorganisms11030788/s1, Table S1: susceptibility tests of four bacterial species isolated from semen extended in Beltsville Thawing Solution with 0.25 mg/mL gentamicin sulfate.

## References

[1] Waberski D., Riesenbeck A., Schulze M., Weitze K.F., Johnson L. (2019). Application of Preserved Boar Semen for Artificial Insemination: Past, Present and Future Challenges. Theriogenology.

[2] AGISAR, WHO (2019). Critically Important Antimicrobials for Human Medicine. https://apps.who.int/iris/bitstream/handle/10665/312266/9789241515528-eng.pdf.

[3] Nitsche-Melkus E., Bortfeldt R., Jung M., Schulze M. (2019). Impact of Hygiene on Bacterial Contamination in Extended Boar Semen: An Eight-Year Retrospective Study of 28 European Ai Centers. Theriogenology.

[4] Morrell J.M., Wallgren M. (2014). Alternatives to Antibiotics in Semen Extenders: A Review. Pathogens.

[5] Schulze M., Nitsche-Melkus E., Hensel B., Jung M., Jakop U. (2020). Antibiotics and Their Alternatives in Artificial Breeding in Livestock. Anim. Reprod. Sci..

[6] Schulze M., Junkes C., Mueller P., Speck S., Ruediger K., Dathe M., Mueller K. (2014). Effects of Cationic Antimicrobial Peptides on Liquid-Preserved Boar Spermatozoa. PLoS ONE.

[7] Speck S., Courtiol A., Junkes C., Dathe M., Müller K., Schulze M. (2014). Cationic Synthetic Peptides: Assessment of Their Antimicrobial Potency in Liquid Preserved Boar Semen. PLoS ONE.

[8] Feugang J.M., Rhoads C.E., Mustapha P.A., Tardif S., Parrish J.J., Willard S.T., Ryan P.L. (2019). Treatment of Boar Sperm with Nanoparticles for Improved Fertility. Theriogenology.

[9] Pérez-Duran F., Acosta-Torres L.S., Serrano-Díaz P.N., Toscano-Torres I.A., Olivo-Zepeda I.B., García-Caxin E., Nuñez-Anita R.E. (2020). Toxicity and Antimicrobial Effect of Silver Nanoparticles in Swine Sperms. Syst. Biol. Reprod. Med..

[10] Barone F., Ventrella D., Zannoni A., Forni M., Bacci M. (2016). Can Microfiltered Seminal Plasma Preserve the Morphofunctional Characteristics of Porcine Spermatozoa in the Absence of Antibiotics? A Preliminary Study. Reprod. Domest. Anim..

[11] Morrell J., Núñez-González A., Crespo-Félez I., Martínez-Martínez S., Alborcia M.-J.M., Fernández-Alegre E., Dominguez J., Gutiérrez-Martín C., Martínez-Pastor F. (2018). Removal of Bacteria from Boar Semen Using a Low-Density Colloid. Theriogenology.

[12] Althouse G., Kuster C., Clark S., Weisiger R. (2000). Field Investigations of Bacterial Contaminants and Their Effects on Extended Porcine Semen. Theriogenology.

[13] Bussalleu E., Yeste M., Sepúlveda L., Torner E., Pinart E., Bonet S. (2011). Effects of Different Concentrations of Enterotoxigenic and Verotoxigenic E. Coli on Boar Sperm Quality. Anim. Reprod. Sci..

[14] Sepúlveda L., Bussalleu E., Yeste M., Bonet S. (2014). Effects of Different Concentrations of Pseudomonas Aeruginosa on Boar Sperm Quality. Anim. Reprod. Sci..

[15] Pinart E., Domènech E., Bussalleu E., Yeste M., Bonet S. (2017). A Comparative Study of the Effects of Escherichia Coli and Clostridium Perfringens Upon Boar Semen Preserved in Liquid Storage. Anim. Reprod. Sci..

[16] Maroto Martín L.O., Muñoz E.C., De Cupere F., Van Driessche E., Echemendia-Blanco D., Rodríguez J.M.M., Beeckmans S. (2010). Bacterial Contamination of Boar Semen Affects the Litter Size. Anim. Reprod. Sci..

[17] Althouse G.C., Lu K.G. (2005). Bacteriospermia in Extended Porcine Semen. Theriogenology.

[18] Úbeda J.L., Ausejo R., Dahmani Y., Falceto M.V., Usan A., Malo C., Perez-Martinez F.C. (2013). Adverse Effects of Members of the Enterobacteriaceae Family on Boar Sperm Quality. Theriogenology.

[19] Schulze M., Ammon C., Rüdiger K., Jung M., Grobbel M. (2015). Analysis of Hygienic Critical Control Points in Boar Semen Production. Theriogenology.

[20] Tvrdá E., Bučko O., Rojková K., Ďuračka M., Kunová S., Kováč J., Benko F., Kačániová M. (2021). The Efficiency of Selected Extenders against Bacterial Contamination of Boar Semen in a Swine Breeding Facility in Western Slovakia. Animals.

[21] Costinar L., Herman V., Pitoiu E., Iancu I., Degi J., Hulea A., Pascu C. (2021). Boar Semen Contamination: Identification of Gram-Negative Bacteria and Antimicrobial Resistance Profile. Animals.

[22] Althouse G., Pierdon M., Lu K. (2008). Thermotemporal Dynamics of Contaminant Bacteria and Antimicrobials in Extended Porcine Semen. Theriogenology.

[23] Gòdia M., Ramayo-Caldas Y., Zingaretti L.M., Darwich L., López S., Rodríguez-Gil J.E., Yeste M., Sánchez A., Clop A. (2020). A Pilot Rna-Seq Study in 40 Pietrain Ejaculates to Characterize the Porcine Sperm Microbiome. Theriogenology.

[24] Johnson L.A., Weitze K.F., Fiser P., Maxwell W.M.C. (2000). Storage of Boar Semen. Anim. Reprod. Sci..

[25] Höfner L., Luther A.-M., Palladini A., Fröhlich T., Waberski D. (2020). Tolerance of Stored Boar Spermatozoa to Autologous Seminal Plasma: A Proteomic and Lipidomic Approach. Int. J. Mol. Sci..

[26] Jäkel H., Scheinpflug K., Mühldorfer K., Gianluppi R., Lucca M.S., Mellagi A.P.G., Bortolozzo F.P., Waberski D. (2021). In Vitro Performance and in Vivo Fertility of Antibiotic-Free Preserved Boar Semen Stored at 5 °C. J. Anim. Sci. Biotechnol..

[27] Luther A.-M., Nguyen T.Q., Verspohl J., Waberski D. (2021). Antimicrobially Active Semen Extenders Allow the Reduction of Antibiotic Use in Pig Insemination. Antibiotics.

[28] Gatti J.-L., Chevrier C., Paquignon M., Dacheux J.-L. (1993). External Ionic Conditions, Internal Ph and Motility of Ram and Boar Spermatozoa. J. Reprod. Fertil..

[29] PPark Y.-J., Shin D.-H., Pang W.-K., Ryu D.-Y., Rahman S., Adegoke E.O., Pang M.-G. (2021). Short-Term Storage of Semen Samples in Acidic Extender Increases the Proportion of Females in Pigs. BMC Veter-Res..

[30] Riesenbeck A., Schulze M., Rüdiger K., Henning H., Waberski D. (2015). Quality Control of Boar Sperm Processing: Implications from European Ai Centres and Two Spermatology Reference Laboratories. Reprod. Domest. Anim..

[31] Schulze M., Jung M., Hensel B. (2022). Science-Based Quality Control in Boar Semen Production. Mol. Reprod. Dev..

[32] Gączarzewicz D., Udała J., Piasecka M., Błaszczyk B., Stankiewicz T. (2016). Bacterial Contamination of Boar Semen and Its Relationship to Sperm Quality Preserved in Commercial Extender Containing Gentamicin Sulfate. Pol. J. Veter- Sci..

[33] Martínez-Pastor F., Lacalle E., Martínez-Martínez S., Fernández-Alegre E., Álvarez-Fernández L., Martinez-Alborcia M.J., Bolarin A., Morrell J.M. (2021). Low Density Porcicoll Separates Spermatozoa from Bacteria and Retains Sperm Quality. Theriogenology.

[34] Schmid S., Henning H., Petrunkina A.M., Weitze K.F., Waberski D. (2013). Response to Capacitating Stimuli Indicates Extender-Related Differences in Boar Sperm Function. J. Anim. Sci..

[35] Drobnis E.Z., Crowe L.M., Berger T., Anchordoguy T.J., Overstreet J.W., Crowe J.H. (1993). Cold Shock Damage Is Due to Lipid Phase Transitions in Cell Membranes: A Demonstration Using Sperm as a Model. J. Exp. Zool..

[36] White I.G. (1993). Lipids and Calcium Uptake of Sperm in Relation to Cold Shock and Preservation: A Review. Reprod. Fertil. Dev..

[37] Schmid S., Henning H., Oldenhof H., Wolkers W.F., Petrunkina A.M., Waberski D. (2013). The Specific Response to Capacitating Stimuli Is a Sensitive Indicator of Chilling Injury in Hypothermically Stored Boar Spermatozoa. Andrology.

[38] Waberski D., Luther A.-M., Grünther B., Jäkel H., Henning H., Vogel C., Peralta W., Weitze K.F. (2019). Sperm Function in Vitro and Fertility after Antibiotic-Free, Hypothermic Storage of Liquid Preserved Boar Semen. Sci. Rep..

[39] Menezes T.A., Mellagi A.P.G., da Silva Oliveira G., Bernardi M.L., Wentz I., Ulguim R.D.R., Bortolozzo F.P. (2020). Antibiotic-Free Extended Boar Semen Preserved under Low Temperature Maintains Acceptable in-Vitro Sperm Quality and Reduces Bacterial Load. Theriogenology.

[40] Jäkel H., Henning H., Luther A.-M., Rohn K., Waberski D. (2021). Assessment of Chilling Injury in Hypothermic Stored Boar Spermatozoa by Multicolor Flow Cytometry. Cytom. A.

[41] Rowe M., Veerus L., Trosvik P., Buckling A., Pizzari T. (2020). The Reproductive Microbiome: An Emerging Driver of Sexual Selection, Sexual Conflict, Mating Systems, and Reproductive Isolation. Trends Ecol. Evol..

