# Supplementary material for: Growth Dynamic and Threshold Values for Spermicidal Effects of Multidrug-Resistant Bacteria in Extended Boar Semen
Source: Microorganisms. 2023 Mar 19;11(3):788. doi: 10.3390/microorganisms11030788 (PMC10058892; doi:10.3390/microorganisms11030788)
Supplement: Supplementary file 1 [file microorganisms-11-00788-s001.zip › microorganisms-2274413-supplementary.pdf]

Supplemental Table S1: Susceptibility test (MIC) of four bacterial species isolated from semen extended in BeltsvilleThawing Solution with 0.25 mg/mL gentamicin sulfate.

	Bacterial species			
Antibiotic	*Serratia marcescens* F/19/002835/M-1	*Klebsiella oxytoca* F/16/001936/M	*Burkholderia cepacia* F/18/003653/M	*Achromobacter xylosoxidans* F/19/002835/M-2
Amoxicillin / Clavulanic acid	8/4	16/8	>= 32/16	8/4
Ampicillin	8	>= 64	>= 32	8
Ceftiofur	>= 8	0,5	>= 8	>= 8
Cephalothin	16	>= 32	>= 32	16
Colistin	2	<= 0,031	>= 4	2
Enrofloxacin	1	>= 8	0,25	1
Erythromycin	>= 8	4	>= 8	>= 8
Florfenicol	4	>= 16	8	4
Gentamicin	>= 16	>= 32	>= 16	>= 16
Penicillin G	>= 16	>= 128	>= 16	>= 16
Spectinomycin	>= 128	>= 16	>= 128	>= 128
Tetracyclin	2	>= 64	>= 16	2
Tiamulin	>= 64	>= 64	>= 64	>= 64
Tilmicosin	>= 32	>= 8/152	>= 32	>= 32
Trimethoprim / Sulfonamid	<= 0,25/4,8	64	1/19	<= 0,25/4,8
Tulathromycin	>= 128	16/8	>= 128	>= 128

Resistance testing is performed by microdilution method according to CLSI Vet01, CLSI Vet01S, CLSI Vet06, CLSI M100S or According to scientifically proven procedures.
The numerical value corresponds to the Minimum Inhibitory Concentration (MIC) of the active substance in µg/ml.
